# On the consequences of intra-operative release versus over-tensioning of the posterior cruciate ligament in total knee arthroplasty

**DOI:** 10.1098/rsif.2024.0588

**Published:** 2024-12-18

**Authors:** Seyyed Hamed Hosseini Nasab, Sabrina Hörmann, Thomas M. Grupp, William R. Taylor, Allan Maas

**Affiliations:** ^1^ Laboratory for Movement Biomechanics, ETH Zürich, Zürich, Switzerland; ^2^ Aesculap AG, Research & Development, Tuttlingen, Germany; ^3^ Ludwig Maximilians University Munich, Department of Orthopaedic and Trauma Surgery, Musculoskeletal University Center Munich (MUM), Campus Grosshadern, Munich, Germany

**Keywords:** total knee arthroplasty, posterior cruciate ligament, musculoskeletal modelling, intra-operative ligament balancing, knee joint mechanics

## Abstract

Intra-operative tensioning of the posterior cruciate ligament (PCL) in total knee arthroplasty (TKA) is commonly based on the surgeon’s experience, resulting in a possibly loose or overly tight PCL. To date, the consequences of different PCL tensioning scenarios for the post-operative biomechanics of the knee remain unclear. Using a comprehensive musculoskeletal modelling approach that allows predictive joint kinematic and kinetic balance, we assessed variations in the movement and loading patterns of the knee as well as changes in ligament and muscle forces during walking in response to systematic variations in the PCL reference strain. The results indicate only small differences in the tibiofemoral and patellofemoral kinematics and kinetics for scenarios involving up to 10% release of the PCL (relative to the baseline reference scenario with 2% residual strain). These observations remain valid for simulations performed with high- as well as with low-conformity implant designs. However, over-tensioning of the ligament was found to considerably overload the tibiofemoral joint, including altered contact mechanics, and may therefore shorten the implant longevity. Finally, no meaningful impact of the PCL reference strain on the muscle force patterns was observed. This study therefore favours balancing the knee with a slightly loose rather than tense PCL, if appropriate intra-operative PCL tension cannot be objectively achieved.

## 1. Introduction

Posterior cruciate-retaining total knee arthroplasty (PCR-TKA) aims to restore the natural knee biomechanics by maintaining the physiological function of the posterior cruciate ligament (PCL) [1,2]. The PCL is the primary restraint to tibial posterior drawer during flexion, probably supporting the physiological femoral rollback mechanism [3–5]. Moreover, femoral posterior translation during flexion is thought to increase the lever arm of the knee extensor mechanism to preserve its mechanical efficiency, thus requiring reduced quadriceps force to extend the knee under load [3,6–8]. Furthermore, advocates of PCR-TKA claim that PCL preservation increases the knee range of motion (ROM) as a physiological femoral rollback precludes early posterior tibiofemoral impingement [7,9]. However, contrary to the proposed benefits of PCL preservation, follow-up studies showed no significant superiority in clinical and functional outcomes of PCR compared with cruciate sacrificing TKAs [10–14]. Specifically, no clinically relevant differences have been found in post-operative ROM and knee stability between PCL retaining and PCL substituting implants [15]. Additionally, researchers have raised concerns about higher inter-subject variability in knee kinematics and even non-physiological paradoxical anterior translation of the femoral condyle among patients with PCR-TKA [7,16–19].

Biomechanical investigations have suggested that improper ligament balancing may contribute to poor outcomes after PCR-TKA [17,20,21]. Specifically, a tight PCL has been associated with excessive femoral posterior translation, high stresses on the polyethylene inlay and limited knee flexion [9,21,22]. Thus, proper tensioning of the PCL has been consistently emphasized as the key to successful PCR-TKA outcomes [23–25]. However, optimal intra-operative PCL balancing is difficult to achieve, particularly since the PCL is short and varies in terms of length and orientation pre- and post-operatively [1,19]. Consequently, partial or complete release of the PCL is commonly performed to prevent possible over-tensioning of the ligament [26–28]. On the other hand, a sacrificed or loose PCL can lead to excessive anterior femoral translation during flexion, increased patellofemoral pressure, knee instability and pain [7,9]. To date, however, very few studies have investigated the influence of PCL tension on knee kinematics and kinetics after PCR-TKA. Since inlay geometry is known to play a key role in joint stabilization, particularly tibiofemoral anterior–posterior (A–P) translation, the interrelationships between PCL tension and inlay constraints are plausibly critical. However, the consequences of cutting the PCL while using low-conformity PCL-retaining implant designs have never been investigated biomechanically.

Due to the technical and ethical challenges of using strain or force sensors to measure PCL tension in living human subjects [29,30], studies have mainly focused on assessing paradoxical implant kinematics using imaging techniques [31–34]. Intra-operatively, this lack of objective assessment approach has meant that adjusting PCL tension through prosthesis size and implantation technique cannot be easily controlled or quantified [16,35–38]. In order to compensate, Donadio *et al*. [35] reported that an oversized femoral component can effectively regulate PCL tension through preventing paradoxical anterior displacement of the femoral condyles during intra-operative passive knee flexion. Notably, their study lacked a post-operative assessment of knee function, particularly concerning PCL loading during weight-bearing activities. The simulation of such loaded knee flexion on multiple cadaveric specimens, however, was able to reveal that PCL strain and tibiofemoral kinematics can both be influenced by varying the implant design and implantation technique [1,3]. In particular, Emodi *et al*. [3] reported that a 4 mm joint line elevation could be correlated with a twofold increase in PCL strain at knee flexion angles exceeding 90°. In order to expand our understanding of the interplay between PCL loading and knee function post-TKA, it is, therefore, crucial to acknowledge that *in vitro* studies are generally limited in their ability to fully replicate the complex physiological loading conditions within the knee.

*In silico* computer modelling is a flexible and efficient approach that can be used to investigate knee joint mechanics after PCR-TKA. While generic musculoskeletal models have a limited capacity to assess the joint contact mechanics and soft tissue loading patterns [39,40], recent advances in musculoskeletal modelling, and in particular, the integration of force-dependent kinematic models within the muscle optimization algorithms, are now able to support more realistic simulation of six degrees of freedom (d.f.) joints that include a detailed representation of the ligaments [41,42]. Using such modelling techniques, previous studies have assessed the influence of implant alignment and collateral ligament strain on post-TKA tibiofemoral contact forces during level walking [43,44]. Nevertheless, the influence of PCL tensioning scenarios on TKA functionality remains unclear.

Using an advanced musculoskeletal modelling framework that can account for the simultaneous contribution of the articular contact, as well as ligament and muscle forces governing post-operative PCR-TKA function, this study aimed to provide a comprehensive understanding of the influence of PCL tensioning on the post-TKA function of regular- as well as low-conformity implants during level walking. Specifically, the goal was to understand the biomechanical consequences of partial, or complete release of the PCL, even in PCL-retaining implant designs.

## 2. Methods

### 2.1. Experimental data

The experimental data used for this study were obtained from the publicly available datasets provided by the *6th Grand Challenge Competition to Predict in vivo Knee Loads* (6th GCC [45]). The datasets include skin-marker motion capture (120 Hz) and ground reaction force (GRF; 1000 Hz) data collected during multiple walking activities performed by a male subject (83 years old, 70 kg, 172 cm) with an instrumented implant (P.F.C. Sigma, DePuy Synthes, Raynham, MA, USA) in his right knee. This implant represents a fixed bearing, unconstrained bicondylar posterior cruciate-retaining design. The motion capture utilized a modified version of the Cleveland Clinic marker set, enhanced with additional markers on the feet and trunk to provide more detailed tracking of lower limb and torso movements [45]. A low- and high-pass Butterworth filter with cut-off frequencies of 6 and 50 Hz was used to smooth the motion-capture data and GRFs. *In vivo* knee contact forces and moments (KCF and KCM, 50 Hz) acting on the tibial tray were also included within the datasets. The forces acting on the medial and lateral sides of the tibial inlay were calculated using the equations provided by the 6th GCC team [46].

### 2.2. Subject-specific knee model

**Figure 1.**
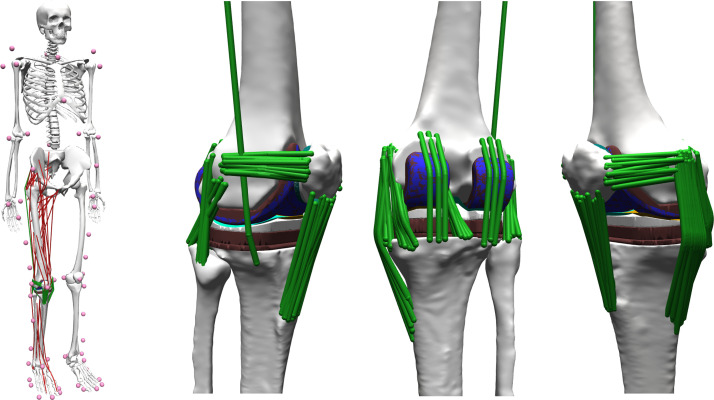
The whole-body musculoskeletal model with detailed representations of the knee structures.

Using the bone geometries reconstructed from CT images and implant CAD geometries provided within the GCC datasets, a detailed knee model was developed enabling the 12 d.f. knee kinematics to be simulated. Implant components were positioned within the tibia and femur using the subject’s bone cuts as the reference*.* An elastic foundation contact model [47] was used to formulate the tibiofemoral and patellofemoral articular contact mechanics. Major knee ligaments including anterolateral and posteromedial bundles of the PCL (aPCL and pPCL), superficial and deep medial collateral ligaments (sMCL and dMCL), lateral collateral ligament (LCL), capsular ligaments (CAPs), medial and lateral patellofemoral ligaments, as well as the iliotibial band were represented by multiple nonlinear springs (figure 1). Attachment sites of the ligaments on the tibia, femur and patella were identified based on guidelines reported in previous cadaveric studies [48–50]. Material properties in general were taken from the literature [5,42,43], with a linear stiffness of 180 N mm^−1^ used for the PCL. Virtual wrapping objects were used to prevent penetration of the ligament fibres into the bones. At 0° knee flexion, a reference strain of 2% was assigned to the PCL to mimic the residual force acting in the healthy knee ligament [29]. Reference strains for other knee ligaments were taken from the literature [43,51].

### 2.3. Baseline musculoskeletal model

A previously developed musculoskeletal (MS) model [42] with 34 d.f. and 40 muscle-tendon units actuating the right lower limb joints was scaled to match the subject’s anthropometry based on skin-marker locations captured in a standing body pose. The lengths of the tibia and femur were further adjusted to fit the bone dimensions extracted from CT images. The detailed knee model was then integrated into the full-body MS model by aligning the subject-specific and generic bone geometries (figure 1).

### 2.4. Gait simulation

Within an inverse kinematics approach, skin-marker trajectories of a representative walking cycle of the subject were used to determine the whole-body kinematics. The obtained primary joint angles and GRF data were then input to the concurrent optimization of muscle activation and kinematics (COMAK [52]) algorithm. This algorithm solves the muscle redundancy problem by simultaneous optimization of articular contact mechanics, muscle and ligament forces, as well as the tibiofemoral and patellofemoral joint kinematics, while minimizing the sum of the squared muscle activations. Subsequently, at each time step, the muscle activations and secondary knee kinematics (including the 5 d.f. of the tibiofemoral and 6 d.f. of the patellofemoral joint) were estimated throughout the entire gait cycle (GC). KCFs/KCMs were normalized to body weight (BW)/body weight multiplied by height (BW ∗ H) and presented against time or the knee flexion angle.

To ensure validity of the modelling framework, the medial and lateral contact forces estimated by the baseline model were compared against the corresponding measured *in vivo* data.

### 2.5. Simulation of different posterior cruciate ligament tensioning scenarios

The baseline reference strain of the PCL (PCL_pre-strain_) was perturbed between −10% (resulting in an almost unloaded ligament throughout the simulation) and +10% (failure strain of the ligament [5]) with 2% intervals. Using the baseline motion-capture and GRF data, the COMAK simulation pipeline was repeated to assess variability in the joint contact mechanics, as well as muscle and ligament loading patterns across the perturbed models.

**Figure 2.**
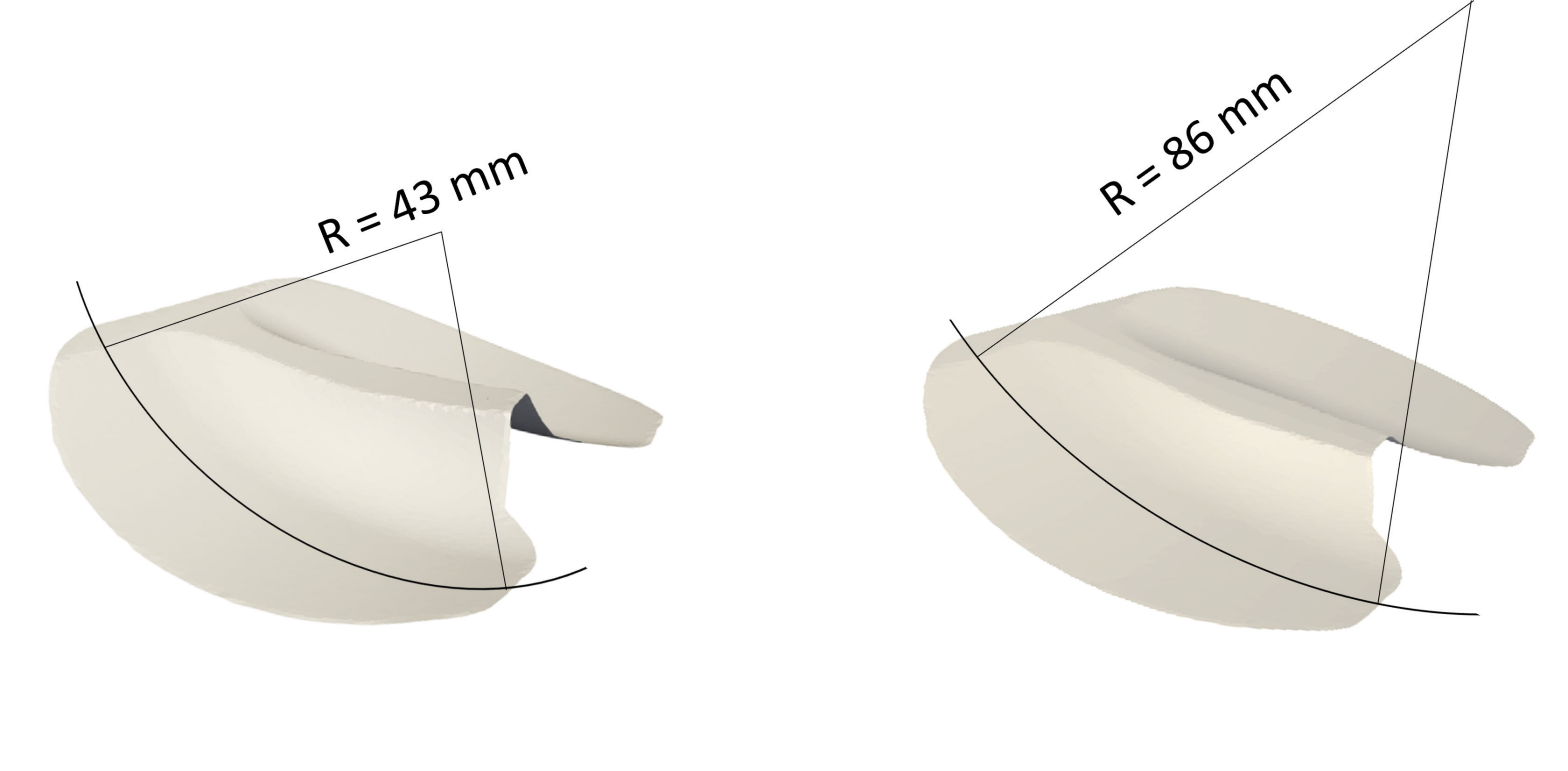
Tibial articular surface of the subject-specific standard-conformity (left) and the simulated low-conformity (right) implant.

To understand the influence of PCL tensioning on post-TKA kinematics and loading conditions within low-conformity implants, the original CAD geometry of the tibial inlay was modified to increase the radius of the articular contact surface (from 43 to 86 mm, figure 2) and thereby reduce the implant conformity. Care was taken to keep the lowest contact points of the mesh unchanged and thereby maintain the joint line at its baseline level, and hence thereby also allow a direct comparison between kinematic and kinetic outcomes. COMAK simulations were repeated for PCL_pre-strain_ perturbing from −10 to +10% and results were compared against those obtained for the subject-specific standard-conformity implant geometry.

## 3. Results

### 3.1. Baseline model results and validation

**Figure 3.**
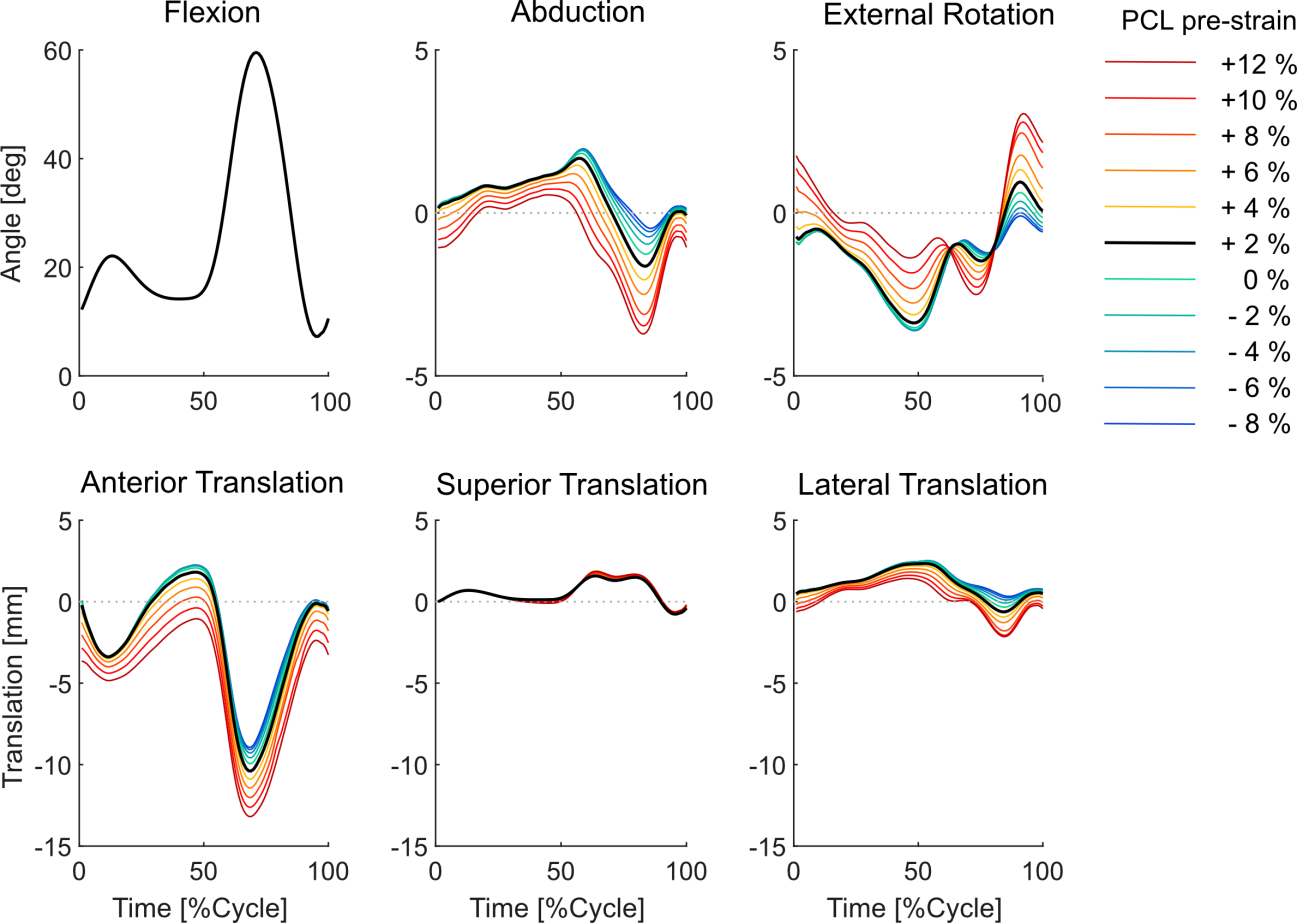
Variations in the tibiofemoral kinematics due to changes in the PCL_pre-strain_.

The baseline model simulation during level walking indicated tibiofemoral flexion angles of up to 57°, while the range of ab-adduction and tibial axial rotation angles were limited to around 3.4° and 5.0°, respectively (figure 3). The anteroposterior translation of the joint showed flexion-dependent characteristics with 11.4 mm maximum posterior translation at around 70% GC.

**Figure 4.**
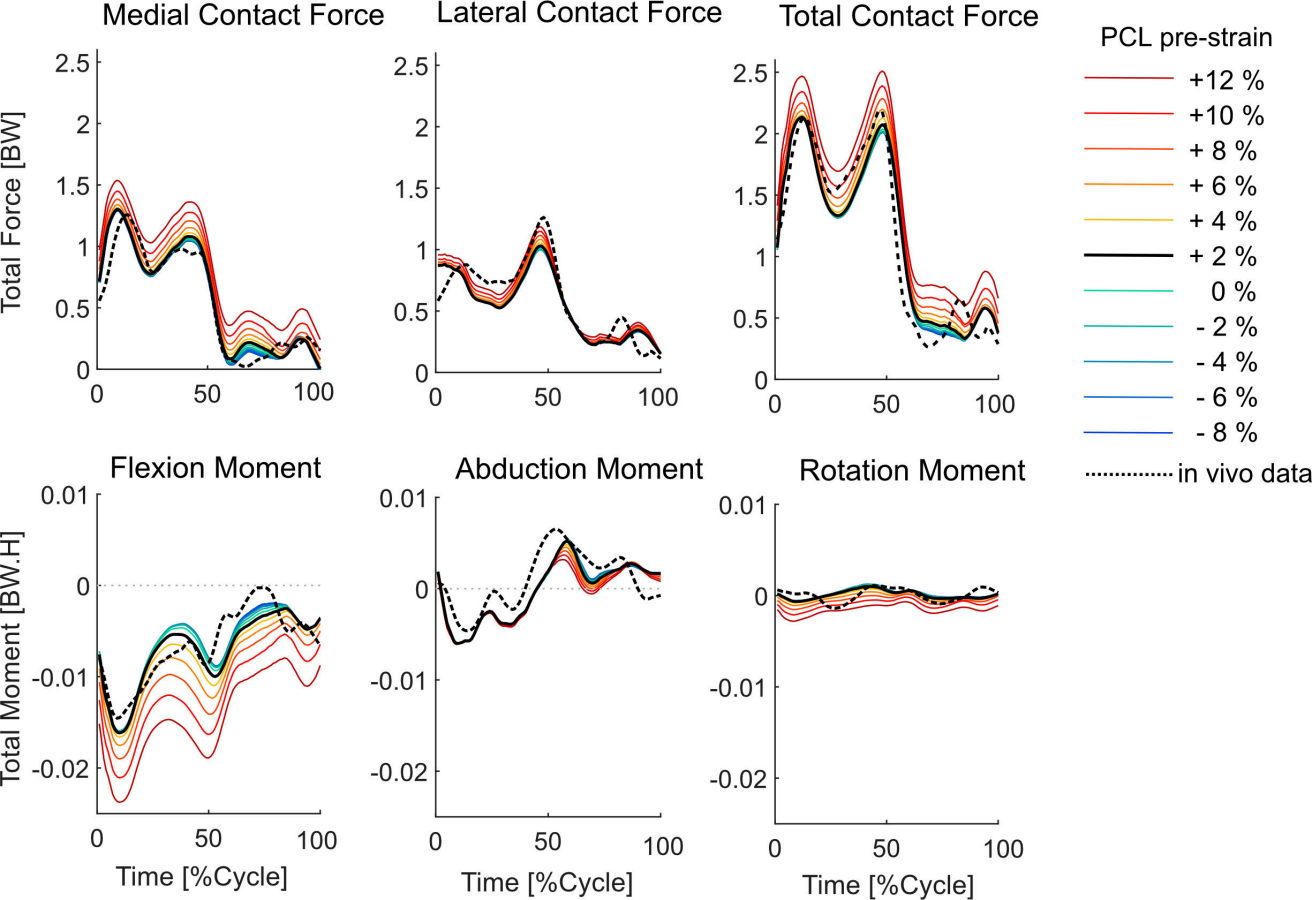
Variations in the tibiofemoral contact forces due to changes in PCL tensioning. *In vivo* forces measured using instrumented implants are plotted as black dashed lines.

Tibiofemoral joint contact forces obtained from the baseline simulation showed very similar trends to those measured *in vivo*, indicating two peaks at around 15% and 50% GC (figure 4). The estimated force magnitudes were also in agreement with the corresponding *in vivo* values (root mean square error (RMSE) 0.21 BW for the total KCF, with RMSE 0.12 BW on the medial, and RMSE 0.14 BW on the lateral condyles). Similarly, the three components of the KCM were well predicted by the modelling framework (with RMSEs of approx. 2.3, 2.6 and 0.9 Nm for the ab-adduction, flexion-extension and axial rotational moments, figure 4).

### 3.2. Impact of different posterior cruciate ligament tensioning scenarios

Varying the PCL_pre-strain_ around its baseline value (2% ± 10%) did not influence the knee flexion angle but altered the adduction and external rotation (by up to 3.5°, figure 3) of the knee joint, mainly during the stance phase. Tightening of the PCL encouraged posterior translation of the femoral relative to the tibial component (3.1 mm at toe-off for 10% PCL tensioning). On the other hand, a loose PCL resulted in a minor anterior femoral translation (around 1 mm at toe-off for 10% decrease in PCL strain, figure 3). The impact of PCL tensioning on SI translation of the tibiofemoral joint was small, but varying the ligament pre-strain from −8 to +12% resulted in 1.8 mm lateral translation of the joint at toe-off (around 63% GC). In general, for the same variation in PCL tensioning, the change in tibiofemoral kinematics due to a loose PCL was less highlighted than those predicted for a tight PCL (e.g. 1 mm anterior translation due to −8%, compared with 3 mm posterior translation due to +12% PCL strain). These findings were generally consistent for simulations using the low-conformity implant; however, the range of variation in the tibiofemoral kinematics was slightly larger for the incongruent implant (cf. Figure 3 versus electronic supplementary material, figure S1).

Increasing the PCL_pre-strain_ strain relative to its baseline value resulted in larger KCFs with a greater impact on the medial than on the lateral KCF (e.g. 0.28 BW increase of the peak force on the medial condyle compared with 0.16 BW increase on the lateral condyle, due to 10% additional PCL tensioning, figure 4). Interestingly, however, loosening of the PCL from its baseline condition did not meaningfully lower the maximum KCF (e.g. less than 0.1 BW due to a 10% reduction in PCL_pre-strain_). The flexion–extension moment was largely affected by changes in the PCL_pre-strain_, whereas the ab-adduction moment was not altered considerably (10.6 Nm change in the flexion–extension moment compared with 1.4 Nm change in the ab-adduction moment across the range of PCL tensioning scenarios, figure 4). Very similar force and moment results were obtained from the simulations performed on the low-conformity implant (electronic supplementary material, figure S2).

**Figure 5.**
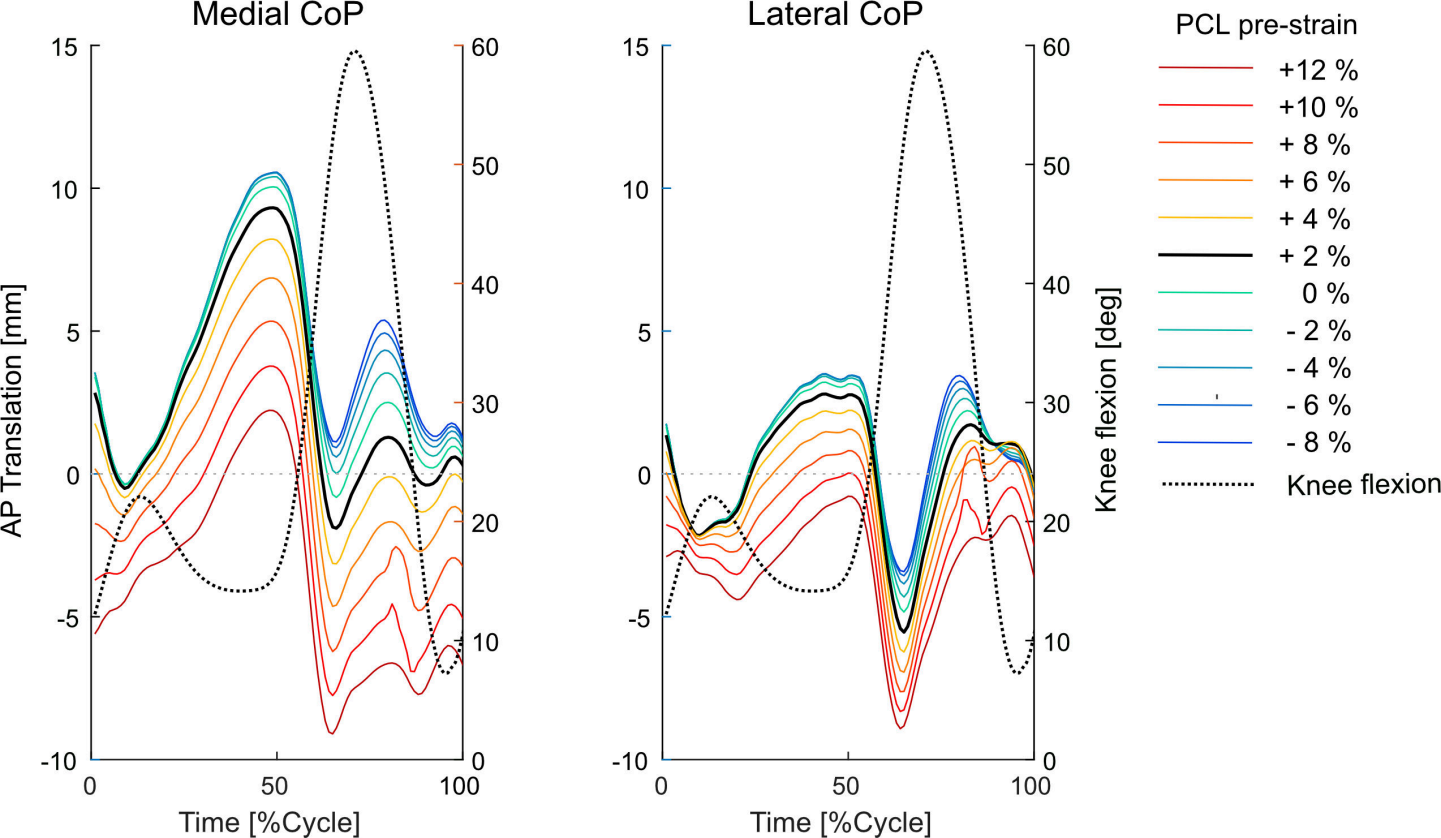
Translation of the CoP on the tibial inlay in the anteroposterior direction due to changes in the PCL_pre-strain_. Implant flexion angle is plotted as dashed black line to assist interpretation of the results.

**Figure 6.**
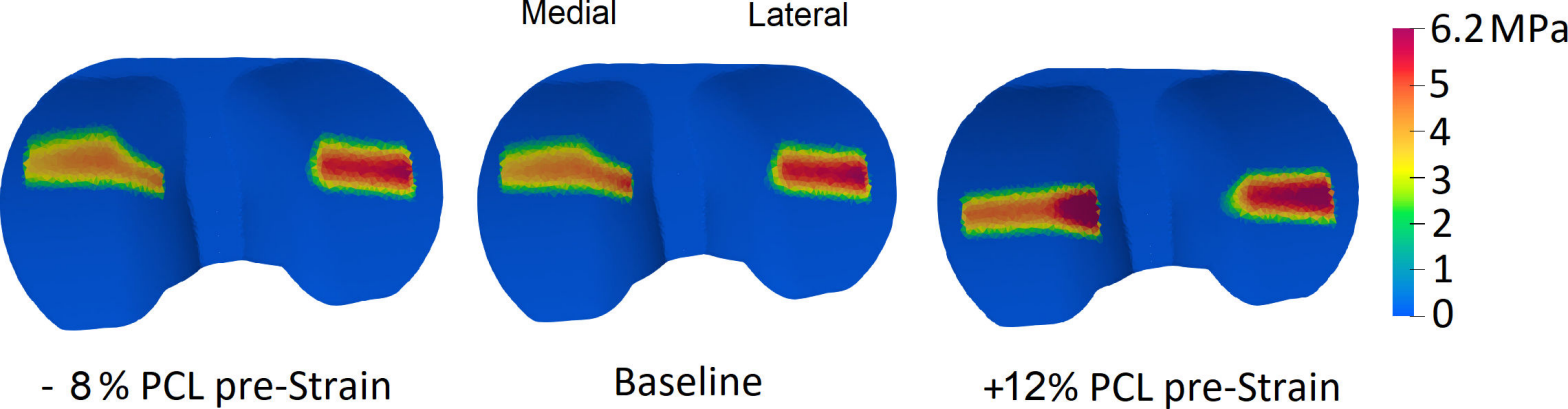
Changes in the tibiofemoral contact pressure map at the occurrence of the second peak of the KCF due to variations in the PCL_pre-strain_.

Compared with the baseline simulation, PCL tensioning scenarios involving a loose PCL generally resulted in the medial and lateral centre of contact pressures (CoPs) on the tibial inlay shifting anteriorly during walking (figures 5 and 6). However, similar to the tibiofemoral kinematic patterns, deviation of the CoP position from its baseline location on the inlay was less highlighted for a more lax than a more tense PCL (e.g. 2 mm anterior shift of the medial CoP for −10% PCL strain compared with 7 mm posterior shift for +10% PCL strain at the instance of the highest KCF). For the low-conformity implant, variations in the position and excursion of the medial and lateral CoPs were larger than those observed for the subject-specific standard-conformity implant (figures 5 and 6 versus electronic supplementary material, figures S3 and S4).

Variation of the PCL_pre-strain_ resulted in only minor changes in the 6 d.f. patellofemoral joint kinematics, with no significant difference between the subject-specific standard-conformity and low-conformity implants (electronic supplementary material, figures S5 and S7). Similarly, the influence of the PCL_pre-strain_ on the patellofemoral contact force was almost negligible (electronic supplementary material, figures S6 and S8).

**Figure 7.**
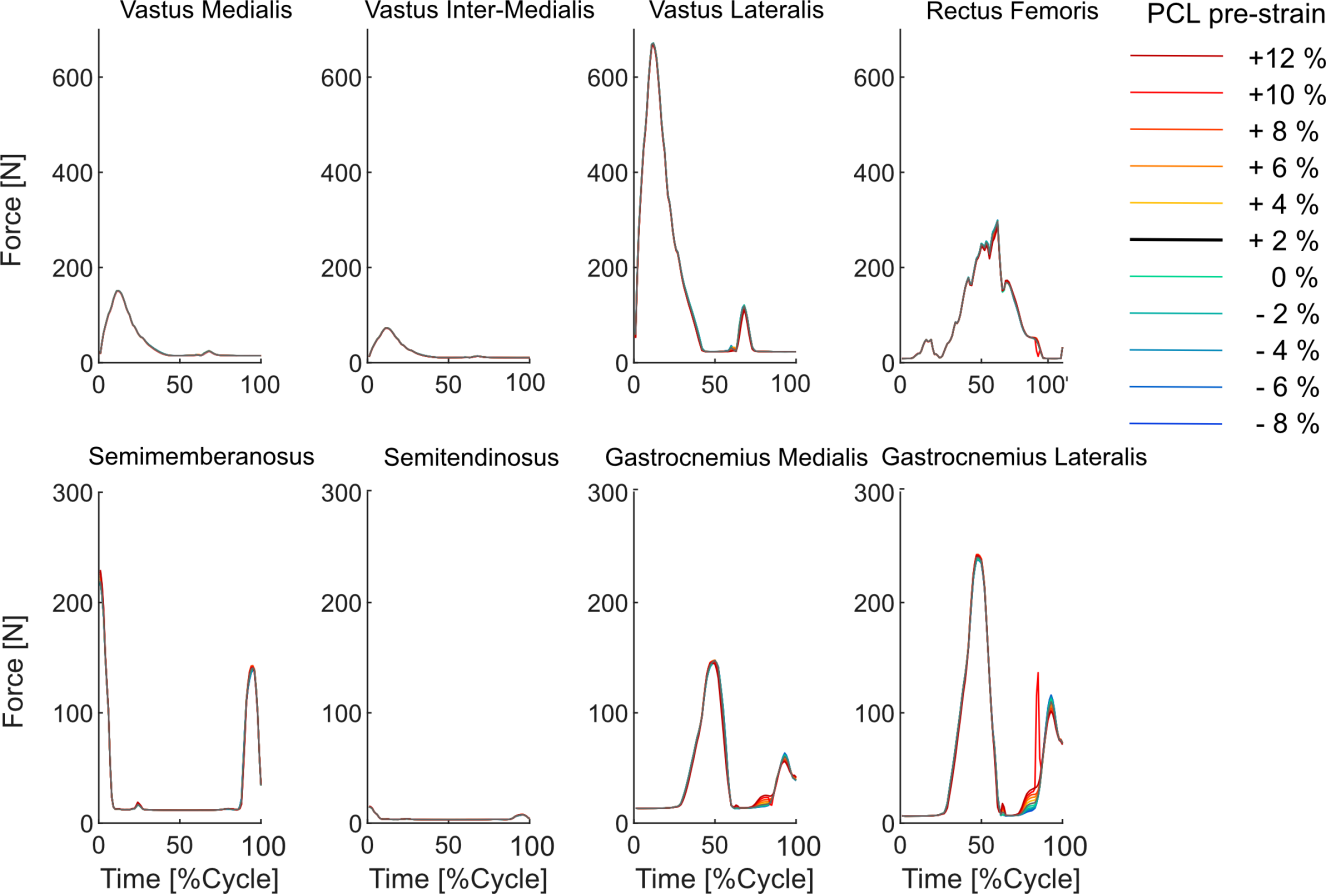
Change of the muscle forces as a result of variation in the PCL_pre-strain_.

**Figure 8.**
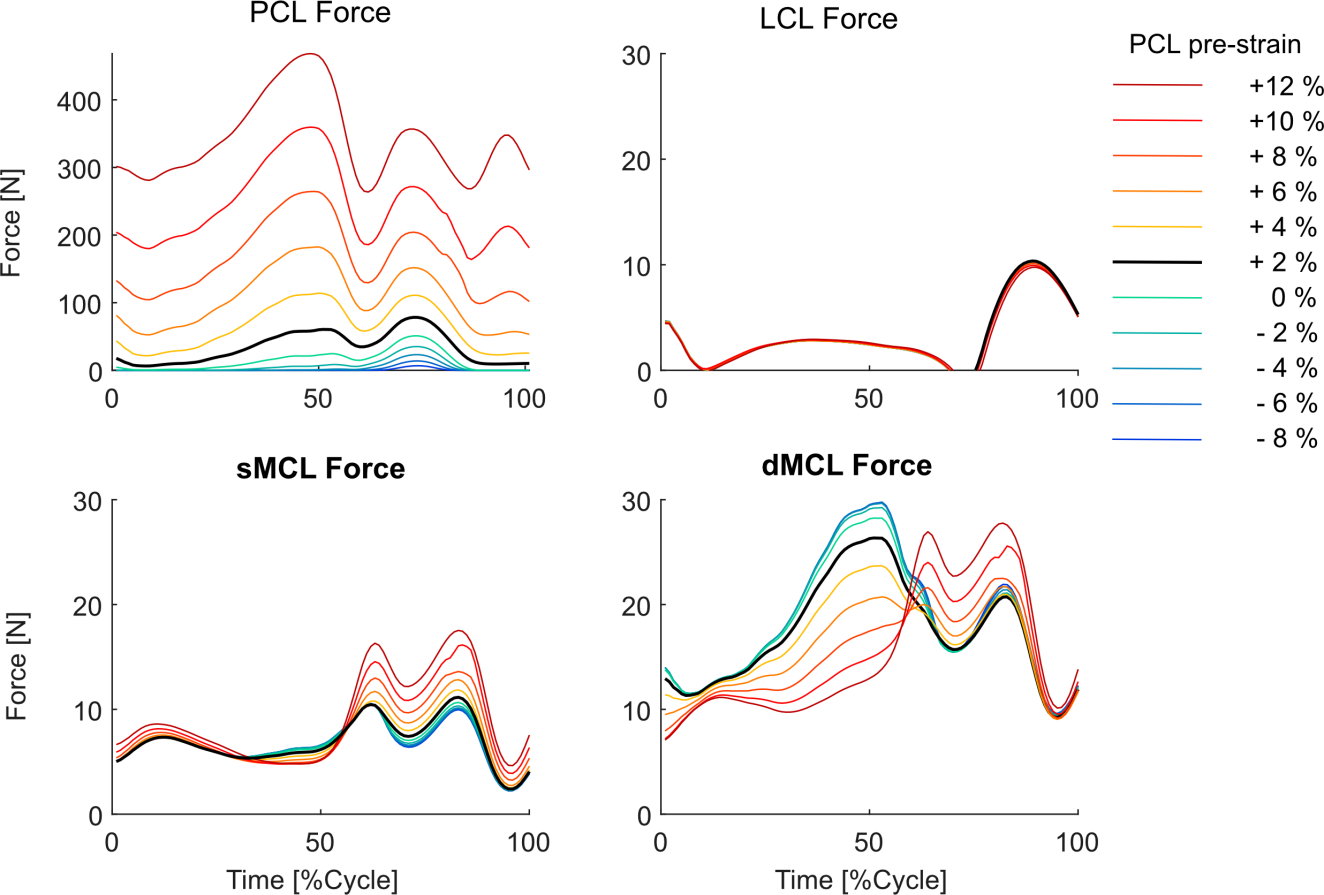
Change of the ligament forces due to variation in the PCL_pre-strain_.

Muscle forces experienced negligible changes due to variations in the PCL_pre-strain_, which was consistent between low-conformity and subject-specific standard-conformity implants (figure 7 and electronic supplementary material, figure S9). However, an increase of the PCL_pre-strain_ resulted in higher PCL forces throughout the entire walking cycle (figure 8). During the stance phase, the sMCL force showed only minor changes, but the dMCL force reduced with increasing PCL_pre-strain_. In the swing phase, both dMCL and sMCL experienced greater forces for scenarios involving higher PCL_pre-strain_. The LCL force remained almost unchanged when different PCL reference strains were tested. In general, compared with the subject-specific standard-conformity implant, simulations performed on the low-conformity implant indicated larger variations in the collateral ligament forces due to changes in the PCL_pre-strain_ (e.g. up to 35 N for the low-conformity versus up to 17 N for the subject-specific standard-conformity inlay, figure 8 versus electronic supplementary material, figure S10).

## 4. Discussion

Despite numerous studies presenting various surgical techniques to improve intra-operative PCL tension in TKA, the relationship between PCL_pre-strain_ and post-operative knee biomechanics, soft tissue loading and muscle activation patterns remains unclear. Using an advanced musculoskeletal modelling framework, we found that the PCL_pre-strain_ can indeed affect movement and loading patterns of the knee during walking. However, an over-tensioned PCL may have more critical drawbacks for the loading conditions within the replaced joint than a loose PCL, and hence also possible consequences for rupture of the ligament itself.

Proper intra-operative tensioning of the PCL has been reported to be technically challenging and surgeons may therefore perform a partial or complete release of the ligament to facilitate gap balancing [26–28,53]. Our results indicate that a partial (or even a complete) release of the PCL should not lead to excessive changes in the knee kinematics during walking. More specifically, during the stance phase of level walking, a 10% release of the PCL_pre-strain_ resulted in only 1–2 mm anterior shift of the tibial medial and lateral CoPs and less than 1° change in the ab-adduction and axial rotation of the knee (figure 3). The minimal impact of a loose PCL on anteroposterior tibiofemoral translation could be attributed to the congruency between the contact surfaces and the steep anterior slope of the standard-conformity insert. Notably, this observation holds true even when low-conformity implants are used (cf. Figure 3 and electronic supplementary material, figure S1). However, our predictive *in silico* approach suggests that over-tensioning of the PCL results in a considerable posterior shift of the femoral component and external rotation of the tibia, with greater effects observed for low-conformity implants (cf. Figures 3 and 5, electronic supplementary material, figures S1 and S3). In addition, an increase in PCL_pre-strain_ was found to increase the KCF (figure 4) and hence possibly reduce longevity of the implant. Since the intra-operative PCL tension is mainly judged based on the clinical experience of the surgeon rather than precise objective measures [28,53], over-tensioning of the PCL is entirely possible. Therefore, to avoid unfavourable consequences of a tense PCL, our findings endorse a slightly loose over a tightly balanced PCL, unless specific intra-operative conditions suggest otherwise.

Advocates of PCR-TKA have proposed that the restored PCL function would encourage femoral rollback and thereby assist the knee extensor mechanism by increasing the muscle moment arms [1,3]. While confirming more posterior translation of the CoPs for scenarios with higher PCL_pre-strain_, our results do not indicate any meaningful impact of PCL_pre-strain_ on the quadriceps and patellar tendon forces during walking (figure 7 and electronic supplementary material, figure S9). Here, potential benefits of the more posterior CoPs for the knee extensor mechanism seem to be compensated by the additional flexion moment generated by a tense PCL (considering its relatively large elevation angle [54]). These findings are supported by a previous *in vitro* study [55] reporting no significant difference between quadriceps force after TKA with cruciate sacrificing or retaining implants and numerous clinical studies [11–14] reporting no superiority of the two implant designs over each other regarding their post-operative functionality.

The interplay between ligaments, serving as passive stabilizers, and muscles, functioning as active stabilizers, is critical for maintaining knee stability [56,57]. Our findings, based on *in silico* modelling of a single subject, suggest that intra-operative PCL tensioning can significantly influence post-operative knee kinematics and loading patterns (figures 3–8). However, variations in muscle strength and neuromuscular control can lead to different responses across patients when it comes to PCL tension balancing strategies. For example, individuals with stronger knee extensor and flexor muscles may better compensate for a looser PCL through increased co-contraction [57,58]. In contrast, those with weaker muscles or higher BWs may face greater instability and functional challenges if the PCL is too loose [56,59]. While our study sheds light on the relative impact of different PCL tensioning conditions (e.g. comparing slight loosening with over-tensioning), future probabilistic modelling studies that account for inter-subject variability in skeletal anatomy and muscle strength could offer deeper insights and lead to more generalized conclusions. Such investigations would help guide surgeons in tailoring intra-operative PCL tensioning strategies to the specific needs and characteristics of each patient.

Our modelling framework estimated a remarkable increase in the medial and lateral KCF as well as a considerable posterior shift of the CoPs induced by higher PCL_pre-strain_ (figures 4 and 5, electronic supplementary material, figures S2, and S3). The observed variations in the joint contact mechanics may accelerate the polyethylene wear and consequently shorten the longevity of the implant. Our findings therefore support previous investigations implicating a tight PCL in posterior tibial wear and polyethylene failure [53,60,61] and highlight the possible consequences of intra-operative over-tensioning of the PCL.

Some limitations of the current study need to be considered. Firstly, we simulated only one GC of level walking for a single subject with a specific implant design. Although factors such as BW, height and muscle strength can vary across the TKA population, potentially affecting absolute joint and ligament forces, we believe these variations do not significantly affect our findings on the relative impact of different PCL tensioning conditions (e.g. comparing slight loosening with over-tensioning) on post-TKA joint mechanics. Moreover, the PCL constraining function may vary across different activities and for distinct implant concepts. While a relatively congruent and a low-conformity implant were used to assess the influence of inlay congruency on our simulations, any generalization of our findings to other implants with very specific functional characteristics should be performed with caution. Secondly, we used linear elements to represent the knee ligaments (including the PCL) with the generic attachment sites mapped onto the subject-specific bone geometries. While this is a common approach in musculoskeletal modelling [30,62–65], it might not be able to mimic the exact biomechanical function of the three-dimensional ligamentous structures. Finally, in the absence of *in vivo* data describing the subject-specific joint kinematics, the baseline musculoskeletal model was validated only against the *in vivo* contact forces measured using an instrumented implant [45]. As a result, the baseline joint movement may differ from the subject-specific implant kinematics. Since our investigations were mainly based on intra-study comparisons, however, the impact of any such deviations should remain negligible.

## 5. Conclusion

The results of our current study indicate that an intra-operative partial release or slight laxity of the PCL may not critically alter the post-operative knee joint mechanics and muscle activation patterns during level walking in subjects undergoing PCR-TKA. In contrast, over-tensioning of the PCL can result in overloading of the tibiofemoral joint and thereby shorten the implant longevity as well as subject the ligament to possible rupture conditions. Therefore, a slightly loose PCL should be favoured over a tightly balanced PCL, unless specific intra-operative conditions indicate otherwise.

## Data Availability

The data used for this *in silico* modelling study are publicly available as part of the datasets provided by the 6th *Grand Challenge Competition to Predict In Vivo Knee Loads* [66]. Furthermore, all data generated during this study is accessible here: [67]. Supplementary material is available online [68].
